# Impact of 23-valent pneumococcal polysaccharide vaccination on non-invasive pneumococcal pneumonia: A regression discontinuity analysis

**DOI:** 10.1017/S0950268826101952

**Published:** 2026-07-23

**Authors:** Femke S. Vernooij, F. Annabel Niessen, Anneke Steens, Mirjam Knol, Hester de Melker, Jan van de Kassteele, C.H. (Henri) Van Werkhoven, PPV23-NIPP Study Group

**Affiliations:** 1 Centre for Infectious Disease Control, National Institute for Public Health and the Environment, Netherlands; 2 Department of Medical Microbiology, Univerisity Medical Center Utrecht, Utrecht University, Netherlands; 3 Julius Center for Health Sciences and Primary Care, Univerisity Medical Center Utrecht, Utrecht University, Netherlands

**Keywords:** pneumococcal infections, pneumonia, pneumococcal polysaccharide vaccine, regression discontinuity design, vaccination impact

## Abstract

The aim of this study was to evaluate the impact of the vaccination programme with 23-valent pneumococcal polysaccharide vaccine (PPV23) on non-invasive pneumococcal pneumonia (NIPP) in adults above 60 years old during the first three post-implementation years. For this retrospective impact study with a regression discontinuity design, we included patients aged 56 to 85 years who presented with NIPP at the hospital between November 1, 2016 and September 30, 2023. NIPP was defined as a positive pneumococcal urinary antigen test without meeting criteria for invasive pneumococcal disease (blood and/or cerebrospinal fluid culture positive for *Streptococcus pneumoniae*). We estimated the overall impact and the impact stratified by time since eligibility for vaccination for each birth cohort. Data were obtained from 14 laboratories throughout the Netherlands, which were estimated to cover 34.9% of the Dutch population. We observed 3821 NIPP patients, of whom 634 were eligible for vaccination. The overall impact was 19.7% (95% confidence interval 8.5, 29.7). Impact estimates decreased over time since eligibility, although non-significantly with overlapping confidence intervals. These insights contribute to the existing body of evidence about PPV23 impact on NIPP, informing future vaccination policy.

## Highlights

- 23-valent pneumococcal polysaccharide vaccine (PPV23) impact in older adults was evaluated.
- Pneumococcal urine antigen test results from 14 laboratories covering 35% of the Netherlands.
- Regression discontinuity design was used for analysis.
- Overall impact of PPV23 on NIPP was 19.7% (95% confidence interval 8.5, 29.7).
- Impact estimates decreased non-significantly over time since eligibility.

## Introduction

*Streptococcus pneumoniae* causes a variety of clinical manifestations, including non-invasive pneumococcal pneumonia (NIPP) and invasive pneumococcal disease (IPD) [1]. In IPD, the bacterium is isolated from a normally sterile body site, and clinical manifestations include bacteraemia and meningitis, which can occur with or without pneumonia. Pneumococcus is the most common cause of community-acquired pneumonia (CAP), posing a major clinical and public health challenge in older adults [2, 3]. The disease burden in adults increases with age [4]. NIPP could be pragmatically defined as pneumococcal CAP not identified as IPD. Approximately 25% of pneumococcal CAP cases become bacteraemic, illustrating how studies focussing on IPD underestimate the pneumococcal disease burden in adults [5].

After implementing the 7-valent pneumococcal conjugate vaccine (PCV) in the Dutch National Immunisation Programme for infants in 2006, and its switch to the 10-valent PCV in 2011, vaccine-type IPD incidence declined in infants and in older adults due to indirect protection [6]. However, non-vaccine-type serotypes emerged, posing a risk especially for older adults. As a result of this serotype replacement, the IPD proportion caused by non-vaccine-type serotypes increased, as did the proportion of NIPP caused by non-vaccine-type serotypes [7]. Changes in overall NIPP incidence remain unclear due to the absence of a national surveillance system. To protect the older population against pneumococcal disease, the 23-valent pneumococcal polysaccharide vaccine (PPV23) was implemented stepwise for adults aged 60 to 79 years from October 2020 onwards. Due to waning immunity [8–12], the Dutch Health Council recommended revaccination every 5 years until the age of 75 years [13]. Birth cohorts were eligible for PPV23 only in 1 year: 1) in 2020, people born between 1941 and 1947 (aged 73 until 79 years at the end of 2020), 2) in 2021, people born between 1948 and 1952 (aged 69 until 73 years at the end of 2021), and 3) in 2022, people born between 1953 and 1956 (aged 66 until 69 years at the end of 2022). The proportion vaccinated among eligible persons was estimated at 73% in 2020 (73 to 79 years), 74% in 2021 (69 to 73 years), and 63% in 2022 (66 to 69 years) [14].

Although PPV23 is known to reduce IPD incidence, vaccine effectiveness (VE) against pneumococcal pneumonia remains inconclusive, with limited data on NIPP despite its substantial disease burden [15, 16]. A meta-analysis by Falkenhorst et al. demonstrated a protective effect of PPV23 against any-serotype pneumococcal pneumonia in adults aged 55 years and older [15]. Restricting to low risk of bias studies, the analysis yielded a pooled VE of 48% (95% confidence interval (CI) 25, 63) from two cohort studies and 53% (95% CI 33, 68) from one case–control study. Contrarily, a more recent meta-analysis by Farrar et al. could not demonstrate a significant protective VE against vaccine-type pneumococcal pneumonia in adults aged 65–74 years (VE 26%, 95% CI −6, 49), with solely one of the individual studies addressing non-bacteraemic cases [16]. VE estimates the effect of vaccination for the individual, but the population effect is reflected by measuring impact of the vaccination programme, estimates of which are not available in the literature. The latter depends on the vaccine uptake and whether vaccine acceptance is associated with baseline infection risk. Therefore, this study aimed to evaluate the impact of the PPV23 vaccination programme on NIPP in the Dutch older adult population during the first three epidemiological years post-implementation. The secondary aim was to estimate birth cohort-specific impact, stratified for time since vaccination eligibility.

## Material and methods

### Study population and design

This retrospective impact study was conducted by the National Institute for Public Health and the Environment (RIVM). Medical microbiology laboratories throughout the Netherlands were approached. They were considered for participation when a pneumococcal urinary antigen test (PUAT) was routinely performed in patients with a clinical presentation suspected of pneumonia at the affiliated hospital(s). The inclusion period was from November 1, 2016 to September 30, 2023, comprising a pre-implementation period (November 1, 2016, until September 30, 2020) and a post-implementation period (November 1, 2020, until September 30, 2023). Patients aged 56–85 years were included.

NIPP was defined as a positive PUAT conducted on a urinary sample during an emergency department visit or hospitalization without meeting criteria for IPD. In case of multiple PUATs during one hospitalization, solely the first positive test was included. The IPD criteria consisted of 1) blood culture positive for *S. pneumoniae* during hospitalization or prior emergency department visit and/or 2) cerebrospinal fluid (CSF) culture positive for *S. pneumoniae* during hospitalization or prior emergency department visit. For some laboratories, it was not feasible to link laboratory data to emergency department and hospitalization data, preventing the identification of the exact period of hospitalization. In these cases, culture data from 5 days before and 5 days after the positive PUAT were considered for the IPD criteria. It was regarded as unlikely that cultures related to suspected pneumonia were performed outside this 10-day timeframe. When no blood and CSF cultures were performed, cases were classified as NIPP. Diagnostic work-up was performed as indicated by clinicians.

### Procedures

Year of birth and microbiological data were obtained from laboratory and/or electronic health care records. Considering the yearly vaccination campaign in October, epidemiological year was defined from November until September. Patients with a positive PUAT in October were excluded from analysis. Age was determined by epidemiological year and year of birth. We evaluated the impact of the vaccination programme in the eligible birth cohorts, defined as the relative reduction of the NIPP incidence in the eligible population. Cases were aggregated by epidemiological year, year of birth, and vaccine eligibility. The population at risk was estimated based on the Dutch population size per year of birth on January 1 of the epidemiological year, adjusted for the national coverage of participating laboratories (Supplementary Material S2).

### Statistical analyses

A regression discontinuity design (RDD) was used to assess the impact of the PPV23 vaccination programme on NIPP in eligible birth cohorts and estimate the number of NIPP cases prevented in the national population (Supplementary Material S3). RDD can be applied for causal effect estimation in observational studies, when a continuous assignment variable (i.e. year of birth) determines intervention eligibility (i.e. PPV23 vaccination programme) [17–19]. The method assumes that individuals just below the cut-off for eligibility (in our case determined by age) have a similar distribution of baseline characteristics compared to individuals just above the cut-off, except for their eligibility for vaccination. The impact of vaccination eligibility can be estimated by comparing the incidence before and after the cut-off. Impact was assessed by comparing the NIPP incidence in the eligible population to the counterfactual NIPP incidence of that population without the vaccination programme in place. The counterfactual was extrapolated from data of ineligible cohorts, both from the pre- and post-implementation period. Note that, because we included 11 months per epidemiological year in the analysis, the incidence estimates need to be interpreted as per 11 months. Since it is commonly assumed that PPV23 does not reduce pneumococcal carriage [20], this method assumed no indirect effects of PPV23 on NIPP incidence (i.e. no herd immunity).

A generalized additive negative binomial regression model was employed to estimate the incidence rate ratio (IRR) between the observed and counterfactual NIPP incidence. Epidemiological year was included in the model as a factor, accounting for yearly variation in pneumococcal epidemiology. To allow a non-linear relation between age and NIPP incidence, age in years was included as a penalized thin plate regression spline. Since the impact is not expected to be the same for all ages, the age spline was separately fitted for the ineligible (pre- and post-implementation years) and eligible populations (post-implementation years), with a common smoothing parameter for the two splines. Restricted maximum likelihood was used for smoothing parameter estimation [21]. To estimate the counterfactual NIPP incidence, the age spline of the ineligible cohort was extrapolated to the eligible cohort. The change in impact over time was examined by including time since PPV23 eligibility in years, assuming a log-linear decrease. Lastly, the logarithm of the study population at risk was used as an offset in the model.

Impact was expressed as relative risk reduction, calculated as: (1 – IRR) x 100%. Due to the interaction between age and vaccine eligibility, direct estimation of the impact from the model parameters is not appropriate [22]. Parametric Monte Carlo (MC) simulations were used to derive impact estimates and 95% CIs. To this end, 100000 random samples were generated from a multivariable normal distribution, using the regression coefficients and variance–covariance matrix of the generalized additive negative binomial model. Point estimates were derived from the 50th percentile of MC simulation results, and 95% CIs were derived from the 2.5th and 97.5th percentiles. Both the overall impact and the impact for each year since PPV23 eligibility per birth cohort were derived. The number of NIPP cases prevented in the national population was estimated from model predictions using the national, age-specific population size. To address underdetection of NIPP, a sensitivity analysis was performed to adjust the number of prevented NIPP cases in eligible birth cohorts, assuming a PUAT sensitivity between 66.6% and 82.3% [23] and a proportion of patients tested between 60% and 80% as a PUAT is not always performed in patients suspected of pneumococcal pneumonia [24].

As a sensitivity analysis, we excluded each laboratory individually to examine its influence on the PPV23 impact estimate. In this analysis, we explored correlations between the impact estimate and the following characteristics: 1) the proportion of NIPP cases in whom culture data was available, 2) the proportion of NIPP cases among all performed PUATs, and 3) the proportion of NIPP cases among all positive PUATs. This analysis was performed since we expect a higher VE of PPV23 against IPD compared to NIPP. If blood and/or CSF cultures are not performed in all pneumonia patients, some IPD cases will be misclassified as NIPP and the impact of PPV23 will be overestimated. Therefore, we hypothesized that the correlation between impact and the proportion of cultures performed would be negative, and when excluding a laboratory with low culture obtainment, the impact should decrease. The second and third analyses both explore whether there was an association between diagnostic work-up (i.e. collecting PUAT in severely ill patients) and the estimated impact. The correlation was estimated with the Spearman’s rank correlation coefficient. All analyses were performed in R (version 4.4.0) [25] using the mgcv package (version 1.9.1) [21].

## Results

**Figure 1.**
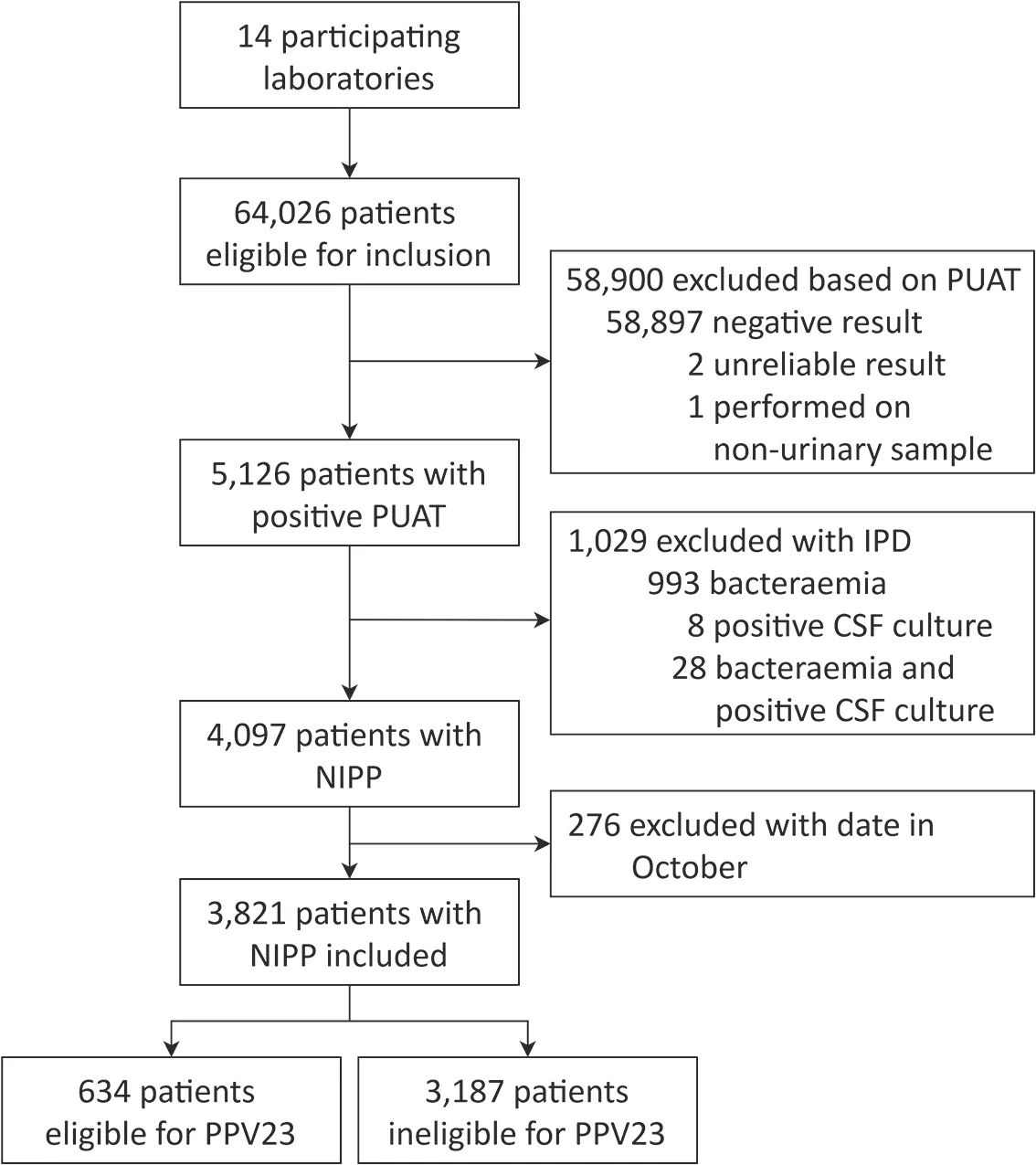
Flowchart of the study population. PUAT = pneumococcal urinary antigen test. IPD = invasive pneumococcal disease. NIPP = non-invasive pneumococcal pneumonia. PPV23 = 23-valent pneumococcal polysaccharide vaccine. Figure 1. long description.

Data were obtained from 14 medical microbiology laboratories throughout the Netherlands, which were estimated to cover 34.9% of the Dutch population (Supplementary Figure S1, Supplementary Table S1). Between November 1, 2016 and September 30, 2023, PUATs were performed in 64026 hospitalizations of patients aged 56 to 85 years in the affiliated hospitals (Figure 1). Of these patients, 5126 (8.0%) had pneumococcal pneumonia confirmed by positive PUAT, including both non-invasive and invasive cases. Blood cultures were performed in 4007 (78.2%) PUAT-positive cases, and 81 (1.6%) had a CSF culture. 1029 (20.1%) of all PUAT-positive cases were classified as IPD and were thus excluded: 993 (19.4%) had bacteraemia, 8 (0.2%) had a positive CSF culture, and 28 (0.5%) had both bacteraemia and a positive CSF culture. The proportion of NIPP cases among those in whom a PUAT was performed, ranged between 3.7% and 8.7% for different epidemiological years (Supplementary Figure S2). After excluding 276 cases occurring in October, a total of 3821 NIPP patients were included for analyses. Apart from absent culture results when cultures were not performed, there were no missing data.

Of the included cases, 2344 occurred during the pre-implementation period (November 2016 until September 2020) and 1477 during the post-implementation period (November 2020 until September 2023, Table 1). The median age of NIPP patients was comparable between these periods: 72 (interquartile range (IQR) 65, 79) years and 71 (IQR 64, 78) years, respectively. Throughout the post-implementation period, 634 (42.9%) cases were eligible for PPV23 vaccination.

**Table 1.** Number of non-invasive pneumococcal pneumonia patients aged 56–85 years included in the study, for the pre-implementation and post-implementation period, by eligibility status Table 1. long description.

	Pre-implementation	Post-implementation		
PPV23 eligibility	Nov 2016 – Sep 2020 (*n* = 2344)	Nov 2020 – Sep 2021 (*n* = 326)	Nov 2021 – Sep 2022 (*n* = 540)	Nov 2022 – Sep 2023 (*n* = 611)
Ineligible	2344 (100)	260 (79.8)	309 (57.2)	274 (44.8)
1 year since eligibility		66 (20.2)^a^	105 (19.4)^b^	80 (13.1)^c^
2 years since eligibility			126 (23.3)^a^	106 (17.3)^b^
3 years since eligibility				151 (24.7)^a^

Data are *n* (%). PPV23 = 23-valent pneumococcal polysaccharide vaccine.

a Birth cohort 1941–1947.

b Birth cohort 1948–1952.

c Birth cohort 1953–1956.

**Figure 2.**
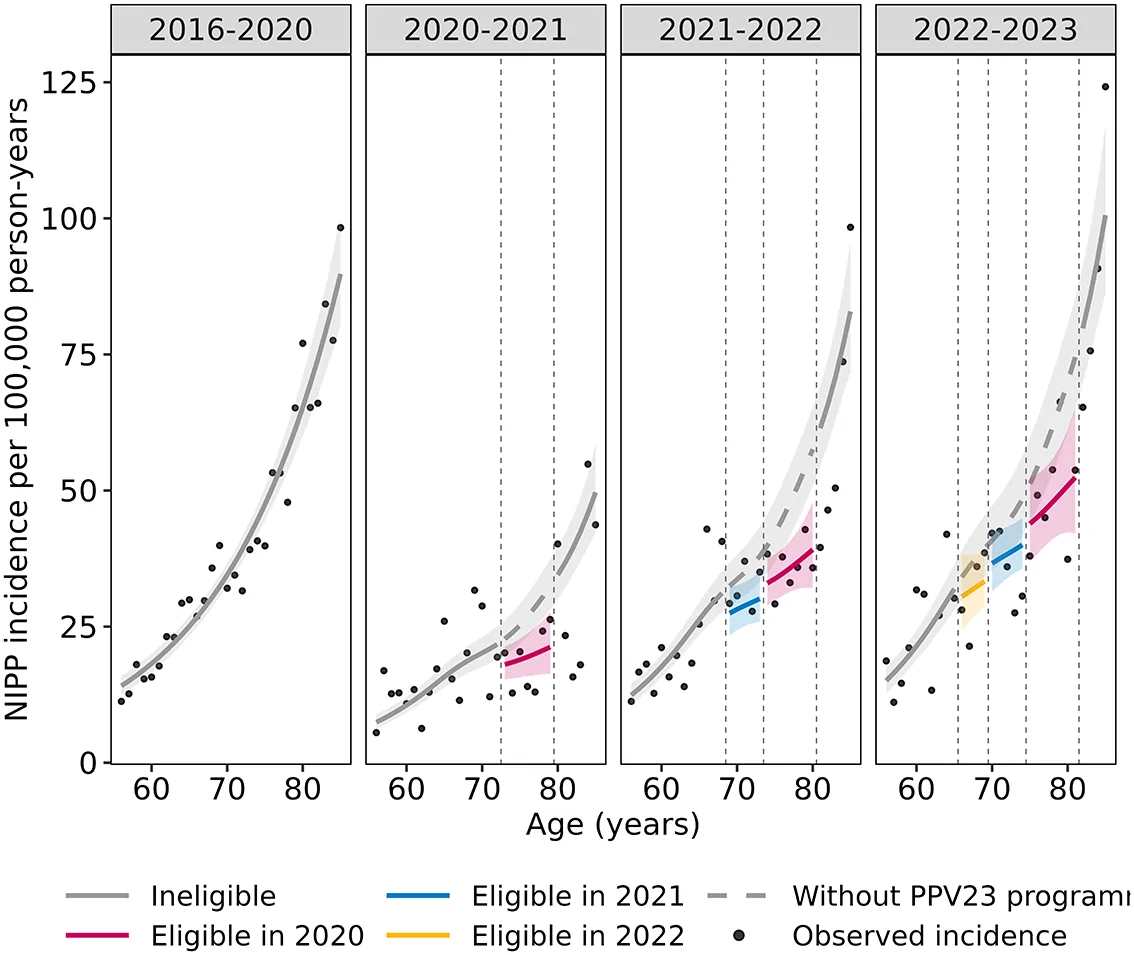
Incidence of non-invasive pneumococcal pneumonia during pre- and post-implementation seasons. Lines represent the modelled incidence and shaded areas the 95% confidence intervals. The colour of the lines shows (the year of) eligibility for PPV23. Note, the incidence is based on data of 11 months per epidemiological year. NIPP = non-invasive pneumococcal pneumonia. PPV23 = 23-valent pneumococcal polysaccharide vaccine. Figure 2. long description.

Figure 2 shows the observed and modelled NIPP incidence for the pre-implementation period and for each post-implementation epidemiological year. For eligible age groups, the counterfactual incidence (i.e. if there would not have been a PPV23 vaccination programme) is also depicted, illustrating the reduced incidence in those cohorts. In general, incidence increased with age and was lower during COVID-19 pandemic seasons (2020–2021 and 2021–2022) compared with pre-implementation years 2016–2020 and post-pandemic year 2022–2023.

**Figure 3.**
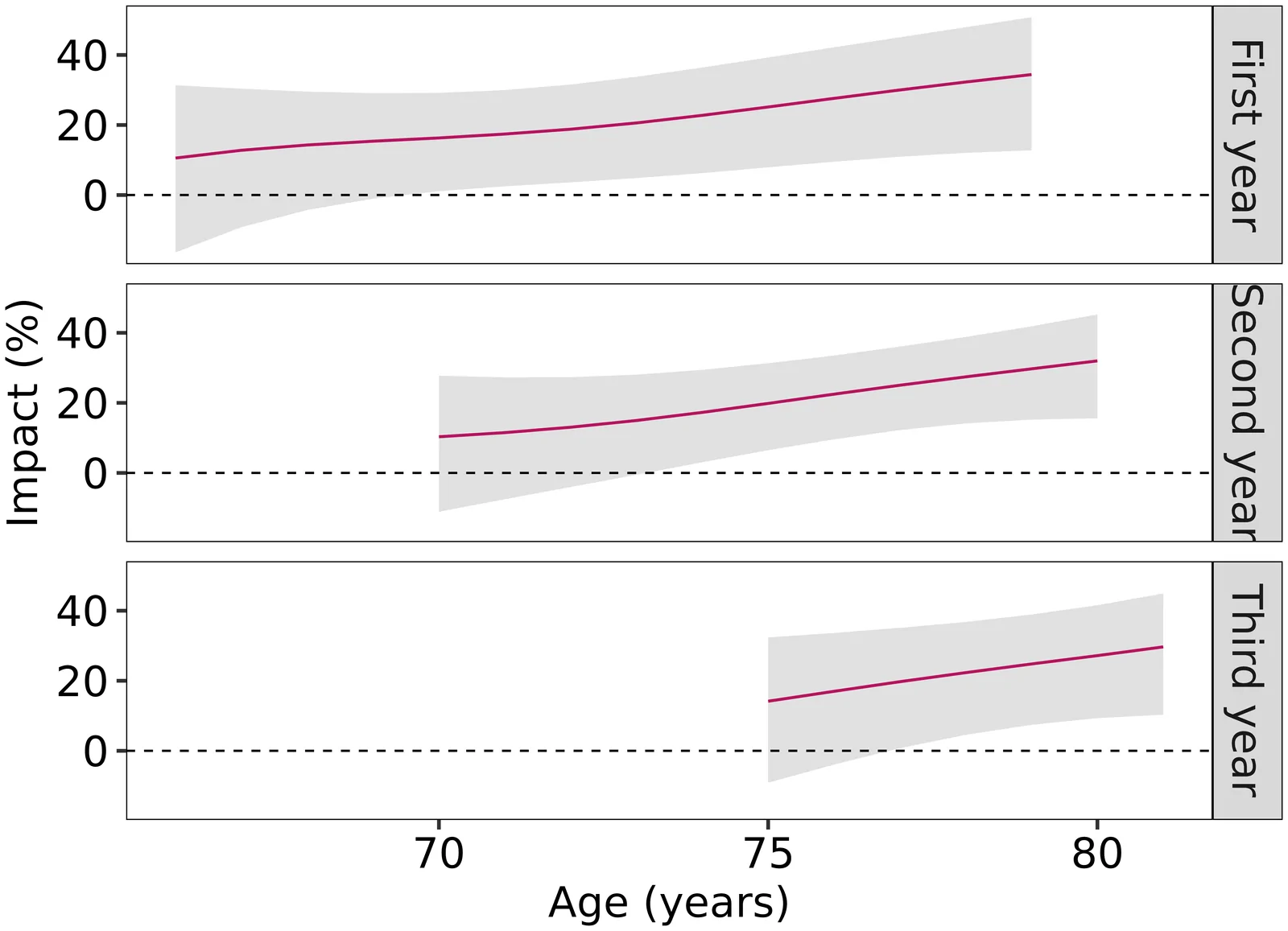
Age-specific impact of the 23-valent pneumococcal pneumonia vaccination programme on non-invasive pneumococcal pneumonia stratified for time since eligibility. Shaded areas represent the 95% confidence intervals. Age-specific estimates are presented in Supplementary Table S4. Figure 3 long description.

After adjustment for age, epidemiological year, and time since eligibility, the overall PPV23 impact for the three post-implementation years was 19.7% (95% CI 8.5–29.7; Table 2). Complete model parameters are presented in Supplementary Table S2 and Supplementary Figure S3, and simulation details in Supplementary Table S3. When stratifying for birth cohort, the impact tended to be higher for the oldest cohort (Table 2). Within birth cohorts, a trend of decreasing impact over time since eligibility was observed, although 95% CIs largely overlapped. A significant impact was observed for the overall period, during the first two post-implementation years, and during the third post-implementation year in the oldest birth cohort. Figure 3 shows age-specific impact estimates stratified by time since eligibility, demonstrating an increasing impact with age. To illustrate, the impact for a 73-year-old individual (i.e. born in 1947) during the first year after PPV23 eligibility was 20.6% (95% CI 4.8–33.8) and decreased to 17.3% (95% CI 3.1–29.4) and 14.2% (95% CI −9.1 to 32.4) in the second and third year, respectively (Supplementary Table S4). The overall number of NIPP prevented in the national population during the epidemiological years was estimated to be 469 (95% CI 186–773; Table 2). When addressing NIPP underdetection, this number increased to a maximum of 1174 (Supplementary Table S5).

**Table 2.** Impact of the 23-valent pneumococcal polysaccharide vaccination programme on the incidence of non-invasive pneumococcal pneumonia in older adults and the estimated number of NIPP cases prevented in the national population Table 2. long description.

Birth cohort	Vaccinated %	Epidemiological year	Impact^a^ % [95% CI]	Incidence^b^		NIPP prevented [95% CI]
				With PPV23 [95% CI]	Without PPV23 [95% CI]	
Overall		2020–2023	19.7 [8.5, 29.7]	33.5 [30.9, 36.4]	41.7 [37.6, 46.3]	469 [186, 773]
1941–1947	73.0	2020–2021	27.1 [10.0, 40.9]	19.3 [16.0, 23.4]	26.5 [23.1, 30.4]	75 [26, 123]
		2021–2022	24.3 [12.3, 34.7]	35.5 [31.4, 40.1]	46.9 [41.2, 53.2]	115 [54, 180]
		2022–2023	21.5 [4.0, 36.0]	47.4 [40.8, 55.1]	60.4 [52.4, 69.7]	127 [21, 237]
1948–1952	74.1	2021–2022	17.8 [3.3, 30.0]	28.8 [25.2, 33.1]	35.1 [30.9, 39.7]	59 [10, 107]
		2022–2023	13.5 [−2.9, 27.4]	38.3 [34.1, 43.1]	44.3 [38.3, 51.3]	56 [−11, 126]
1953–1956	63.0	2022–2023	13.3 [−6.9, 29.5]	31.9 [26.7, 38.1]	36.8 [32.1, 42.2]	40 [−19, 96]

a Adjusted for time since eligibility, age, and epidemiological year.

b Incidence per 100000 person-years. CI = confidence interval. PPV23 = 23-valent pneumococcal polysaccharide vaccine. NIPP = non-invasive pneumococcal pneumonia.

Individual laboratories contributed between 1.5% to 14.5% of the NIPP cases. In the leave-one-laboratory-out sensitivity analysis, the overall impact estimate ranged between 15.2% and 24.0%, all demonstrating a significant effect (Supplementary Figure S4). Contradictory to our hypothesis, a positive correlation was observed between the point estimate and the proportion of NIPP patients in whom a culture was performed (correlation coefficient 0.6, *p*-value 0.029; Supplementary Figure S5). However, we did not find an explanation for this, hence we ascribe this finding to chance. Other correlations were not statistically significant.

## Discussion

This study aimed to evaluate the PPV23 vaccination programme impact on NIPP in the Dutch older adult population during the first three epidemiological years after implementation. Our findings demonstrated a significant protective overall impact of 19.7%, taking time since eligibility, age, and epidemiological year into account. Point estimates were lower over time since eligibility within birth cohorts spanning multiple seasons; however, 95% CIs largely overlapped. NIPP incidence varied across epidemiological years, substantially influencing the number of NIPP cases prevented. For instance, although the impact in the oldest birth cohort was higher during the first year since eligibility for the programme compared with the second year, the number of prevented NIPP was lower due to the lower baseline incidence in the first COVID-19 pandemic year.

When estimating vaccination programme impact rather than VE, several factors should be considered while interpreting results. Impact measures the effect in the entire population eligible for the programme, encompassing both vaccinated and unvaccinated individuals, whereas VE focuses solely on the effect in vaccinated persons [26]. Since PPV23 is considered to cause merely direct effects (i.e. lack of herd immunity), the impact assesses the direct protection in the eligible individuals compared to the counterfactual scenario in which there would not have been a vaccination programme. Notably, the extent of this impact is strongly affected by the proportion of vaccinated individuals within the population. As this proportion differed across birth cohorts (73.0%, 74.1%, and 63.0%), direct comparisons between impact estimates cannot be interpreted as differences in VE estimates. As we do not have information about whether the vaccine uptake depended on certain (medical) factors, we did not attempt to estimate the VE by combining the impact estimates and vaccine coverage, as to avoid bias. However, if we would assume that people accepting the vaccine have the same baseline risk of NIPP as people not accepting the vaccine, an impact of 20% with a vaccine uptake of 70% would translate into a VE of 29% for any-serotype NIPP, which is in line with estimates found in the literature.

Although a direct comparison is not possible, our findings of a protective PPV23 effect on NIPP are in line with studies estimating the VE of PPV23 against similar outcomes. A Spanish cohort study assessed the VE against non-bacteraemic pneumococcal pneumonia (NBPP) within 5 years post-vaccination in adults aged 60 years and older [10]. The analysis included 72 NBPP cases and obtained a reduced hazard in vaccinated individuals (HR 0.5, 95% CI 0.3, 0.9), corresponding to a VE of approximately 50%. In a test-negative case–control study conducted in Japan, Suzuki et al. found a VE of 27.4% (95% CI 3.2–45.6) in adults aged 65 years and older [12]. They included both non-invasive and invasive pneumococcal pneumonia cases (*n* = 419), although 97% concerned NBPP cases. In stratified analyses, the authors observed a non-significant trend of decreasing VE with both increasing age and time since vaccination, which resembles what we observed in the impact estimates over time. Two case–control studies conducted in the Republic of Korea did not observe a protective VE against NBPP in adults aged 65 years and older [27, 28]. A matched case–control study conducted by Kim et al. included 557 NBPP cases and found a VE of 10.2% (95% CI −15.1 to 30.0) [28]. When restricting to adults aged 65 until 74 years, the VE increased to 35.0% (95% CI 2.3–56.7). Using a test-negative design, Heo and colleagues found a VE of −0.1% (95% CI −43.7 to 30.2), with similar findings stratified for age (1.4% for adults aged 65–74 years and −3.8% for adults aged 75 years and above) [27]. As the study included only 161 NBPP cases, the authors acknowledged the insufficient statistical power. Notably, the study periods of aforementioned studies varied in time since the implementation of paediatric PCV vaccination programmes. The proportion of NIPP that is caused by PPV23 serotypes will decrease when indirect effects of paediatric programmes increase. The indirect effects of the paediatric programmes may not have been fully established at the time of conducting the studies, while during our study period, these indirect effects were already in place.

We observed a lower impact of PPV23 in younger age groups. Based on the literature presented above and the hypothesis that immunosenescence decreases the VE with increasing age, one may expect the opposite. However, the proportion of vaccinated persons was substantially lower in the youngest birth cohort. Furthermore, the impact also depends on the proportion of NIPP cases caused by PPV23 serotypes, which differs by age. We do not have information on the serotype distribution and whether this may also partly explain the difference in impact. Moreover, the decreased impact in younger age groups may also be due to chance, as suggested by the wide 95% CIs.

One strength of our study was that the NIPP incidence estimation was based on laboratory-confirmed diagnosis rather than relying on International Classification of Diseases (ICD) codes. ICD codes are not assigned by clinicians in the Netherlands, are not necessarily based on diagnostic testing, and are therefore less specific. Lower specificity will lead to less precise and likely smaller impact estimates. Additionally, compared to other observational studies evaluating PPV23 protection against NBPP, the sample size of 3821 NIPP cases was relatively large. Thereby, our study achieved coverage of over a third of the Netherlands. Furthermore, the leave-one-laboratory-out sensitivity analysis showed similar impact estimates, which makes it more likely that the participating laboratories are representative for the medical microbiology laboratories in the Netherlands. Another strength was the assessment of impact stratified for time since eligibility. Finally, applying the RDD provided a robust framework for causal effect estimation in observational studies [18].

Our study also has some limitations. Firstly, as pneumococcal vaccination status in adults is not nationally registered in the Netherlands, information about the proportion of vaccinated individuals among ineligibles in our study population was not available. If this proportion increased after the vaccination programme was implemented due to increased vaccination awareness, this could have lowered the NIPP incidence among ineligible birth cohorts. Extrapolating this incidence to the eligible counterfactual situation would lead to an underestimated impact in the eligible birth cohorts. In fact, such incidence reduction among ineligible birth cohorts should be considered part of the vaccination programme impact, but the regression discontinuity model does not incorporate this. Based on data covering over 98% of Dutch public pharmacies [29], pneumococcal vaccine dispensation was 0.3% in ineligible adults 55–85 years of age in 2020–2022, compared to 0.1% in prior years, with a maximum of 0.9% in those aged 80 years in 2020 (courtesy of *Stichting Farmaceutische Kengetallen*). This indeed not only confirms an increase in ineligible age groups but also reassures that absolute bias will be minimal. National reimbursement data available at GIPdatabank [30] confirmed that the proportion of vaccinated ineligibles was small. Secondly, NIPP patients in whom a PUAT was not performed could not be included in our study, resulting in an underestimation of the incidence and prevented cases. As illustrated by the sensitivity analysis, the incidence was further underestimated due to the moderate PUAT sensitivity [23]. After adjusting for the sensitivity and assuming that a PUAT was performed in 70.0% of NIPP patients, the number of NIPP prevented among eligible birth cohorts increased from 469 to 906, illustrating the possible magnitude of underestimation. However, since the incidence is non-differentially underestimated for both ineligibles and eligibles, the relative impact is unlikely to be influenced. Thirdly, the impact could have been further underestimated due to the imperfect test specificity of PUAT. While its specificity is reported to be high (97.2%), this may be overestimated as culture is used as the reference standard, which itself has limited specificity [23]. Besides, in low-prevalence settings like ours, even a small decrease in test specificity can lead to a substantial number of false positives relative to true positives, especially in combination with a moderate sensitivity. Fourthly, the generalisability of our findings may be limited to more severe NIPP cases, as performing a PUAT is only recommended in severely ill CAP patients in the Dutch guidelines [24]. Lastly, we included pre-implementation epidemiological years to effectively model the relation between age and NIPP incidence for the counterfactual scenario, applying a consistent association across years. The assumption of a constant age-incidence relation may have been compromised during the first two post-implementation seasons, which coincided with the COVID-19 pandemic. Stricter adherence to social distancing measures by eligible older age groups could have further reduced their incidence compared to their counterfactual incidence, as the latter was based on the pre-implementation and pre-pandemic age-incidence relation. Although model diagnostics did not indicate significant residual deviations between epidemiological years (data not shown), this could have potentially overestimated the impact in older age groups.

The need for better insight into the NIPP disease burden and PPV23 impact was highlighted by a recent cost-effectiveness study evaluating pneumococcal vaccination strategies for older adults in the Netherlands [31]. Sensitivity analyses showed that without a protective effect against NIPP hospitalization, none of the pneumococcal vaccination strategies proved to be cost-effective, highlighting the importance of gaining insight into pneumococcal vaccination effects against NIPP. Due to the lack of data for that study, NIPP incidence was calculated from the total number of all-cause pneumonia ICD codes used in hospitals, assuming that 22.1% was attributable to *S. pneumoniae*, based on the prevalence observed in the CAPITA trial [32]. By providing real-world NIPP incidence data, our study can provide valuable insights for such cost-effectiveness analyses. Researchers can utilize current findings to validate their simulations by comparing the modelled disease burden with our estimates.

## Conclusions

In conclusion, the PPV23 vaccination programme provided an impact of 19.7% against NIPP in the Dutch older adult population during the first 3 years post-implementation. The decreasing impact over time, although non-significant, was consistent with the prevailing understanding of waning immunity after PPV23 vaccination. These insights contribute to the evidence about PPV23 impact on NIPP available for informing future vaccination policy-making.

## Supporting information

10.1017/S0950268826101952.sm001 Vernooij et al. supplementary material Vernooij et al. supplementary material

## Data Availability

Motivated requests for data and scripts required to replicate the results can be directed to the corresponding author. Data sharing may be subject to restrictions because of privacy regulations.

## Supplementary material

The supplementary material for this article can be found at http://doi.org/10.1017/S0950268826101952.

## Prior publication statement

A preliminary version of this manuscript was published as part of the PhD thesis of the 2^nd^ author in Niessen, A 2025, ’Monitoring and preventing severe acute respiratory infections: a focus on COVID-19 and pneumococcal disease’, UMC Utrecht. https://doi.org/10.33540/2786. Available at http://hdl.handle.net/1874/457142.

## Long descriptions

### [Figure 1] Long description

A total of 14 laboratories contributed 64026 PUAT results, of which 5126 patients had a positive PUAT. 1029 of these were excluded because they had invasive pneumococcal disease (IPD) and another 276 because they were tested in October, leaving 3821 patients with non-invasive pneumococcal pneumonia (NIPP) for the anaysis. Of these, 634 had been eligible for the 23-valent pneumococcal polysaccharide vaccine (PPV23).

Navigate back to [Figure 1].

### [Table 1] Long description

A total of 2344 non-invasive pneumococcal pneumonia (NIPP) cases were observed between November 2016 and September 2020, all being ineligible for pneumococcal vaccination. Between November 2020 and September 2021, 326 NIPP cases were obserevd, of which 66 (20.2%) were in their first year since eligibility (birth cohort 1941–1947). Between November 2021 and September 2022, 540 NIPP cases were observed, of which 105 (19.4%) were in their first year since eligibility (birth cohort 1948–1952) and 126 (23.3%) were in their second year since eligiblity (birth cohort 1941–1947). Between November 2022 and September 2023, 611 NIPP cases were obserevd, of which 80 (13.1%) were in their first year since eligibility (birth cohort 1953–1956), 106 (17.3%) were in their second year since eligibility (birth cohort 1948–1952), and 151 (24.7%) were in their third year since eligibility (birth cohort 1941–1947).

Navigate back to [Table 1].

### [Figure 2] Long description

The figure has 4 panels, all showing the incidence of non-invasive pneumococcal pneumonia as a function of age for different periods. The x-axis shows age in years, ranging from 55 to 85, and the y-axis shows incidence per 100,000 person-years. The observed incidences are visualized as dots while the modelled incidence is visualized as a line with a shaded 95% confidence area. Panel one displays the pre-vaccination epidemilogical years 2016–2017 to 2019–2020, all aggregated to one curve; panel two to four displays the epidemiological years 2020–2021, 2021–2022, and 2022–2023, respectively. Each panel shows the same trend of increasing incidence as a function of age in the unvaccinated age groups. The vaccinated age groups consistently show a lower incidence compared to their expected incidence.

Navigate back to [Figure 2].

### [Table 2] Long description

In the birth cohort 1953–1956 that were offered PPV23 at the start of the epidemiological year 2022–2023, vaccination uptake was 63.0%. In the epidemiological year 2022–2023, impact in the birth cohort 1953–1956 was estimated at 13.3 [95% CI −6.9, 29.5], with an estimated incidence under the PPV23 programme of 31.9 [95% CI 26.7, 38.1], compared to 36.8 [95% CI 32.1, 42.2] without the programme. The number of prevented NIPP cases was estimated at 40 [95% CI -19, 96].

In the birth cohort 1948–1952 that were offered PPV23 at the start of the epidemiological year 2021–2022, vaccination uptake was 74.1%. In the epidemiological year 2021–2022, impact in the birth cohort 1948–1952 was estimated at 17.8 [95% CI 3.3, 30.0], with an estimated incidence under the PPV23 programme of 28.8 [95% CI 25.2, 33.1], compared to 35.1 [95% CI 30.9, 39.7] without the programme. The number of prevented NIPP cases was estimated at 59 [95% CI 10, 107]. In the epidemiological year 2022–2023, impact in the birth cohort 1948–1952 was estimated at 13.5 [95% CI −2.9, 27.4], with an estimated incidence under the PPV23 programme of 38.3 [95% CI 34.1, 43.1], compared to 44.3 [95% CI 38.3, 51.3] without the programme. The number of prevented NIPP cases was estimated at 56 [95% CI −11, 126].

In the birth cohort 1941–1947 that were offered PPV23 at the start of the epidemiological year 2020–2021, vaccination uptake was 73.0%. In the epidemiological year 2020–2021, impact in the birth cohort 1941–1947 was estimated at 27.1 [95% CI 10.0, 40.9], with an estimated incidence under the PPV23 programme of 19.3 [95% CI 16.0, 23.4], compared to 26.5 [95% CI 23.1, 30.4] without the programme. The number of prevented NIPP cases was estimated at 75 [95% CI 26, 123]. In the epidemiological year 2021–2022, impact in the birth cohort 1941–1947 was estimated at 24.3 [95% CI 12.3, 34.7], with an estimated incidence under the PPV23 programme of 35.5 [95% CI 31.4, 40.1], compared to 46.9 [95% CI 41.2, 53.2] without the programme. The number of prevented NIPP cases was estimated at 115 [95% CI 54, 180]. In the epidemiological year 2022–2023, impact in the birth cohort 1941–1947 was estimated at 21.5 [95% CI 4.0, 36.0], with an estimated incidence under the PPV23 programme of 47.4 [95% CI 40.8, 55.1], compared to 60.4 [95% CI 52.4, 69.7] without the programme. The number of prevented NIPP cases was estimated at 127 [95% CI 21, 237].

Overall, in the epidemiological years 2020–2021 to 2022–2023, the impact was 19.7% [95% confidence interval (CI) 8.5, 29.7], with an estimated incidence per 100000 person years under the PPV23 programme of 33.5 [95% CI 30.9, 36.4] compared to 41.7 [95% CI 37.6, 46.3] in the counterfactual scenario without the programme. The overall number of prevented NIPP cases in the three epidemiological years was estimated at 469 [95% CI 186, 773].

Navigate back to [Table 2].

### [Figure 3 Long description

It has 3 panels, the first one showing impact in the first year after vaccination eligiblity and using data from all three epidemiological years, the second one showing impact in the second year after vaccination eligibility and using data from the second and third epidemiological years, and the third one showing impact in the third year after vaccination eligibility and using data from the last epidemiological year only. The impact is visualized as a line for the point estimate and shading for the 95% confidence area. Data underlying this graph are provided in Supplementary Table S4.

Navigate back to [Figure 3.
