# Supplementary material for: Impact of 23-valent pneumococcal polysaccharide vaccination on non-invasive pneumococcal pneumonia: A regression discontinuity analysis
Source: Epidemiol Infect. 2026 Jul 23;154:e115. doi: 10.1017/S0950268826101952 (PMC13735529; doi:10.1017/S0950268826101952)
Supplement: Supplementary file 1 [file S0950268826101952sup001.pdf]

Supplementary appendix

Supplement to: Vernooij FS et al. Impact of the 23-valent pneumococcal polysaccharide vaccination programme against non-invasive pneumococcal pneumonia in the Dutch older adult population: a regression discontinuity analysis.

PPV23-NIPP study group names and affiliations

Wouter van den Bijllaardt	Microvida, Laboratory of Medical Microbiology, Amphia Hospital, Breda, The Netherlands
Bjorn Lars Herpers	Regional Public Health Laboratory Kennemerland, Haarlem
Michael P.M. van der Linden	IJsselland Ziekenhuis, Capelle aan den IJssel
Michel Linsen	Meander Medisch Centrum Amersfoort
Maaike S.M. van Mourik	Department of Medical Microbiology and Infection Control, University Medical Center Utrecht
Alewijn Ott	Certe, department of Medical Microbiology, Groningen, The Netherlands
Wouter Rozemeijer	Noordwest Ziekenhuisgroep
Maarten J Schijffelen	Laboratory for Medical Microbiology and Public Health, Hengelo, The Netherlands
Carla van Tienen	Maasstad hospital
Peter C. Wever	Department of Medical Microbiology and Infection Control, Jeroen Bosch Hospital, ’s-Hertogenbosch, The Netherlands

Supplemental 1. Strengthening the reporting of observational studies in epidemiology (STROBE) checklist

	Item No	Recommendation	Manuscript section
**Title and abstract**	1	(*a*) Indicate the study's design with a commonly used term in the title or the abstract	Title and abstract
		(*b*) Provide in the abstract an informative and balanced summary of what was done and what was found	Abstract
**Introduction**			
Background/ rationale	2	Explain the scientific background and rationale for the investigation being reported	Introduction
Objectives	3	State specific objectives, including any prespecified hypotheses	Introduction, paragraph 3
**Methods**			
Study design	4	Present key elements of study design early in the paper	Methods, study population and design, paragraph 1
Setting	5	Describe the setting, locations, and relevant dates, including periods of recruitment, exposure, follow-up, and data collection	Methods, study population and design
Participants	6	(*a*) Give the eligibility criteria, and the sources and methods of selection of participants. Describe methods of follow-up	Methods, study population and design
		(*b*) For matched studies, give matching criteria and number of exposed and unexposed	N/A
Variables	7	Clearly define all outcomes, exposures, predictors, potential confounders, and	Methods, study population and design, and procedures

		effect modifiers. Give diagnostic criteria, if applicable	
Data sources/ measurement	8*	For each variable of interest, give sources of data and details of methods of assessment (measurement). Describe comparability of assessment methods if there is more than one group	Methods, study population and design, and procedures
Bias	9	Describe any efforts to address potential sources of bias	Supplemental 3
Study size	10	Explain how the study size was arrived at	N/A
Quantitative variables	11	Explain how quantitative variables were handled in the analyses. If applicable, describe which groupings were chosen and why	Methods, statistical analyses
Statistical methods	12	(*a*) Describe all statistical methods, including those used to control for confounding	Methods, statistical analyses, supplemental 2, and supplemental 3
		(*b*) Describe any methods used to examine subgroups and interactions	Methods, statistical analyses, paragraph 2 and 3
		(*c*) Explain how missing data were addressed	Methods, study population and design, paragraph 2
		(*d*) If applicable, explain how loss to follow-up was addressed	N/A
		(*e*) Describe any sensitivity analyses	Methods, statistical analyses, paragraph 3 and 4
**Results**			
Participants	13*	*(a)* Report numbers of individuals at each stage of study—eg numbers potentially eligible, examined for eligibility, confirmed eligible, included in the study, completing follow-up, and analysed	Results, paragraph 1

		(b) Give reasons for non-participation at each stage	Results, paragraph 1
		(c) Consider use of a flow diagram	Figure 1
Descriptive data	14*	*(a)* Give characteristics of study participants (eg demographic, clinical, social) and information on exposures and potential confounders	Results, paragraph 2, and table 1
		(b) Indicate number of participants with missing data for each variable of interest	Results, paragraph 1
		(c) Summarise follow-up time (eg, average and total amount)	N/A
Outcome data	15*	Report numbers of outcome events or summary measures over time	Results, paragraph 1 and 2, and table 1
Main results	16	(*a*) Give unadjusted estimates and, if applicable, confounder-adjusted estimates and their precision (eg, 95% confidence interval). Make clear which confounders were adjusted for and why they were included	Unadjusted estimates are not presented, as such estimates would not provide meaningful insights given the high variability in incidence across different seasons and ages.<br><br>Results, paragraph 4, and supplemental 5-7
		(*b*) Report category boundaries when continuous variables were categorized	N/A
		(*c*) If relevant, consider translating estimates of relative risk into absolute risk for a meaningful time period	Results, paragraph 4, and table 3
Other analyses	17	Report other analyses done—eg analyses of subgroups and interactions, and sensitivity analyses	Results, paragraph 5, and supplemental 8 and 9
**Discussion**			

Key results	18	Summarise key results with reference to study objectives	Discussion, paragraph 1
Limitations	19	Discuss limitations of the study, taking into account sources of potential bias or imprecision. Discuss both direction and magnitude of any potential bias	Results, paragraph 4 and 5, discussion, paragraph 6, supplemental 2, assumptions and limitations, and supplemental 3, paragraph 2
Interpretation	20	Give a cautious overall interpretation of results considering objectives, limitations, multiplicity of analyses, results from similar studies, and other relevant evidence	Conclusion
Generalisability	21	Discuss the generalisability (external validity) of the study results	Discussion, paragraph 5
**Other information**			
Funding	22	Give the source of funding and the role of the funders for the present study and, if applicable, for the original study on which the present article is based	N/A

*Give information separately for exposed and unexposed groups. N/A=not applicable or not available.

Supplemental 2. Population at risk

Since catchment areas of Dutch laboratories are not readily available, the population at risk was estimated based on the national coverage of the participating laboratories and the national population size.

National coverage of participating laboratories

Dutch medical microbiology laboratories have been voluntarily submitting invasive pneumococcal disease (IPD) isolates for all age groups to the Netherlands Reference Laboratory for Bacterial Meningitis (NRLBM) for serotyping since 2017. Based on this national IPD surveillance data available at the National Institute for Public Health and the Environment (RIVM), we calculated the proportion of the total IPD cases aged between 56 and 85 years that was submitted by the participating laboratories. This proportion, referred to as the coverage, served as a proxy for the relative catchment area of the included laboratories.

To preclude age-dependent deviations in the number of pneumococcal isolates sent in to the NRLBM caused by either the COVID-19 pandemic or the vaccination programme with the 23-valent pneumococcal polysaccharide vaccine (PPV23; started autumn 2020), we included solely pre-pandemic and pre-implementation epidemiological years to determine the coverage. Hence, data from November 1, 2017, to September 30, 2019, were included. After excluding cases from October and the case from the Caribbean Netherlands, 7,015 IPD cases were included. Dates were based on culture dates, when available. Missing culture dates (n=199, 2.8%) were imputed with the date of sample receipt by the NRLBM, adjusted for the median difference in days between culture date and date of receipt (5 days; interquartile range 4, 6). IPD cases were categorised as originating from either laboratories participating in our study or from non-participating laboratories. As two laboratories only provided non-invasive pneumococcal pneumonia (NIPP) data for a part of the inclusion period, the estimated coverage of the participating laboratories varied across periods. We obtained data from 12 laboratories for the period from November 2016 until January 2017 (first period), from 13 laboratories for February 2017 until September 2020 (second period), from 14 laboratories for November 2020 until September 2022 (third period), and from 13 laboratories for November 2022 until 2023 (fourth period).

To examine whether the national coverage of participating laboratories varied with age, logistic regression generalised additive models were fitted for each period. The proportion of IPD cases originating from participating laboratories was estimated with two models: a model with a penalised thin plate regression spline for age in years (age model) and a model with only an intercept (intercept model). Restricted maximum likelihood was applied for smoothing parameter estimation. The

intercept model was favoured if it showed either a decrease in Akaike Information Criterion (AIC) or if the increase in AIC was two points or less. Significance level was set at 0.05 and 95% Wald confidence intervals (CIs) were obtained.

Age-specific coverage estimates are shown in supplemental figure 1. Age did not significantly improve the estimates (supplemental table 1). Therefore, coverages estimated by the intercept model were applied: 34.5% for period 1, 34.7% for period 2, 34.9% for period 3, and 31.9% for period 4.

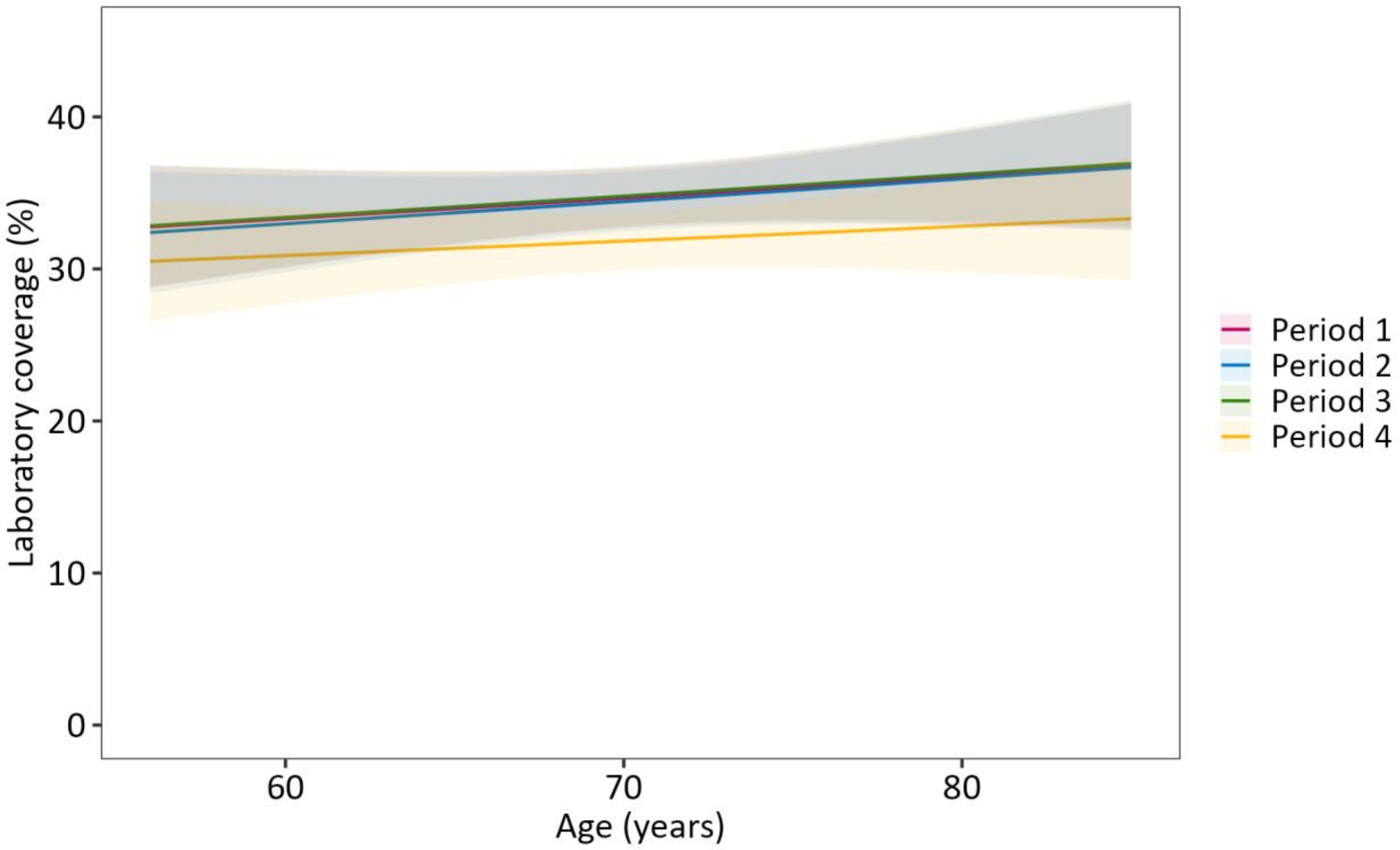

Supplemental figure 1: National coverage of participating laboratories for the different periods by age
The coverage was based on the proportion of invasive pneumococcal disease cases in the Netherlands for whom the isolates were submitted by the participating laboratories. Period 1: November 2016 to January 2017 (n=12 laboratories). Period 2: February 2017 to September 2020 (n=13 laboratories). Period 3: November 2020 to September 2022 (n=14 laboratories). Period 4: November 2022 to September 2023 (n=13 laboratories). Shaded areas represent the 95% confidence intervals.

Supplemental table 1

National coverage of participating laboratories per study period derived by the generalised additive models as described in the supplementary methods

	Age model			Intercept model	
	Coverage* % [95% CI]	Age (years) p-value†	AIC	Coverage % [95% CI]	AIC
Period 1	34.5 [32.6, 36.5]	0.248	2,945	34.5 [32.6, 36.5]	2,944
Period 2	34.7 [32.8, 36.7]	0.284	2,952	34.7 [32.8, 36.7]	2,951
Period 3	34.9 [32.9, 36.8]	0.271	2,955	34.9 [32.9, 36.8]	2,954
Period 4	31.9 [30.0, 33.8]	0.445	2,862	31.9 [30.0, 33.8]	2,860

Period 1: November 2016 to January 2017 (n=12 laboratories). Period 2: February 2017 to September 2020 (n=13 laboratories). Period 3: November 2020 to September 2022 (n=14 laboratories). Period 4: November 2022 to September 2023 (n=13 laboratories). *=coverage estimate averaged over all ages. †=approximate p-value for the null hypothesis that the smooth term is zero. CI=confidence interval. AIC=Akaike Information Criterion.

Adjusted national population size

For each epidemiological year, age-specific national population sizes on January 1 were obtained from Statistics Netherlands.[1] Each population size was adjusted with the corresponding laboratory coverage. For seasons 2017-2018 until 2021-2022, the coverage of the second period was applied, and the coverage of the third period was used for season 2022-2023. As one laboratory provided partial data for epidemiological year 2016-2017, the population size was adjusted with the weighted average of the coverage estimates for the first and second period. The weighting was based on the proportion of IPD cases received by the NRLBM from November to January and from February to September, respectively.

Assumptions and limitations

By applying the aforementioned method to estimate the population at risk, a few assumptions have been made. Firstly, the proportion of IPD cases reported by the laboratories was assumed to be representative of the underlying catchment area, without systematic reporting differences between participating and non-participating laboratories. Secondly, we assumed that the catchment areas were consistent over time, as we applied constant coverage estimates for each epidemiological year. Furthermore, by using coverage estimates based on data until September 2019, we assumed that the catchment area did not significantly change during the remainder of the study period.

Our method has some limitations. Because of the retrospective nature of our study, follow-up data on the study participants was not available. Therefore, we did not take into account deaths of study participants, potentially overestimating the population at risk. By applying the population size on January 1 each year, the population at risk was slightly underestimated for the months November and December and slightly overestimated for January until September. However, since this is non-

differential (i.e. equivalent for the eligible and ineligible cohorts), this does not affect the vaccine impact estimates.

Supplemental 3. Rationale for and approach to the regression discontinuity design

The regression discontinuity design (RDD) provides a robust approach that can be utilised for causal effect estimation, when random intervention assignment is unfeasible, which is the case in observational research.[2-4] In the RDD framework, individuals are either exposed or unexposed to an intervention based on a threshold rule using a continuous variable with a predefined cut-off (assignment variable). Therefore, when intervention assignment is based solely on such threshold, baseline characteristics are randomly distributed around that cut-off. As eligibility for vaccination programmes is often determined by year of birth, RDD is a useful method for evaluating national immunisation programme's impact directly at the eligibility threshold. Since adults aged 66 to 79 years at time of vaccination were eligible for PPV23 during our study period, we used two predefined eligibility cut-offs in our RDD analysis. Therefore, instead of estimating the effect at the thresholds, the impact in the whole eligible group was assessed. This was done by modelling the counterfactual (i.e. the NIPP incidence in absence of the PPV23 immunisation programme in the eligible birth cohorts), by extrapolating the incidence in the ineligible birth cohorts. Notably, the RDD estimates the impact of direct effects of vaccination.[2] As PPV23 vaccination is assumed to provide only direct protection and no indirect protection via herd immunity,[5] RDD is a suitable approach.

Several assumptions of the RDD must be met for reliable causal effect estimation based on real-world data.[2-4] Firstly, the exposure must be allocated based on the assignment variable. Secondly, the measurement of this assignment variable is continuous. Thirdly, continuity of the assignment variable at the cut-off is required, as discontinuity can indicate manipulation of the assignment variable. Since our exposure (eligibility for the PPV23 immunisation programme) is determined by year of birth, a continuous variable that cannot be manipulated for an individual, it is likely that the second and third assumption were met in our RDD analysis. However, a small proportion of ineligible individuals were vaccinated with PPV23, violating the first assumption. Regarding this proportion of 0.3% to be neglectable based on data covering over 98% of Dutch public pharmacies[6] and on the reimbursement data from the GIPdatabank,[7] the impact estimate of the vaccination programme is unlikely to be significantly influenced by this. The fourth assumption is that individuals just below and above the threshold are similar (i.e. exchangeable) with regard to baseline covariables. This exchangeability assumption can be assessed by demonstrating balance of patient characteristics between the exposure groups.[3; 4] As additional patient data were not obtained, we were unable to verify the exchangeability assumption. Nonetheless, as eligibility for the PPV23 vaccination programme is merely based on year of birth, we can reasonably assume that the eligible and ineligible individuals around the cut-offs were comparable regarding other risk factors. Additionally, as proposed by Oldenburg et al. when using wider bandwidths around the cut-off, the assignment

variable can be included as a flexible functional form.[3] Therefore, since NIPP incidence increases with age, we included age (derived from the assignment variable year of birth) as a spline to account for the increasing risk, enhancing the likelihood that the exchangeability assumption was met.

Supplemental 4. Non-invasive pneumococcal pneumonia stratified for epidemiological year

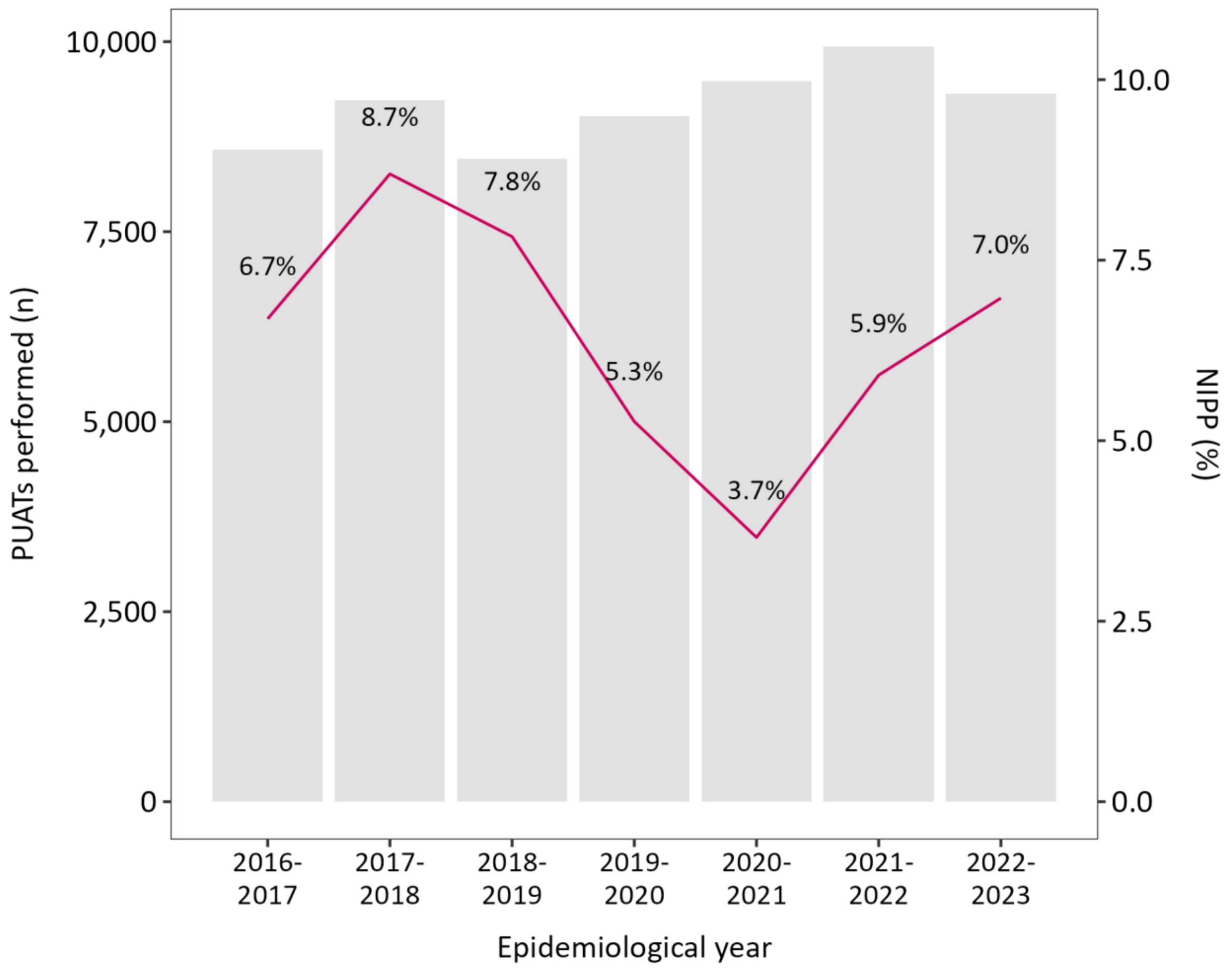

Supplemental figure 2: Number of pneumococcal urinary antigen tests performed in the hospitals affiliated to the participating laboratories (bars) and the proportion of non-invasive pneumococcal pneumonia cases (red line) per epidemiological year

PUAT=pneumococcal urinary antigen test. NIPP=non-invasive pneumococcal pneumonia, which was defined as having a positive PUAT and no blood and cerebrospinal fluid culture positive for *Streptococcus pneumoniae*.

Supplemental 5. Model parameters

Supplemental table 2

Complete model parameters of the negative binomial regression model

Parametric coefficients	Coefficient	Standard error	p-value*
Intercept	-7.997	0.045	<0.001
Epidemiological year			
2016-2017 (reference)	-	-	-
2017-2018	0.321	0.060	<0.001
2018-2019	0.104	0.062	0.092
2019-2020	-0.288	0.067	<0.001
2020-2021	-0.515	0.076	<0.001
2021-2022	-0.003	0.070	0.968
2022-2023	0.191	0.077	0.014
PPV23 eligibility	-0.243	0.154	0.114
Time since eligibility (years)	0.068	0.083	0.412
**Smooth terms**		**edf†**	**p-value‡**
Interaction of age (years) with			
PPV23 ineligibility#		3.730	<0.001
PPV23 eligibility#		1.498	0.267

The impact estimate of 19.7% (95% CI 8.5, 29.7) is derived by Parametric Monte Carlo simulation and is the sum of the direct effect of PPV23 eligibility and the age-PPV23 eligibility interaction effect. *=p-value based on Wald test. †=effective degrees of freedom of smoothing terms (degree of complexity). ‡=p-value based on approximate chi-square test. #=see supplemental figure 3 for the relationship between age and the log-incidence. PPV23=23-valent pneumococcal polysaccharide vaccine.

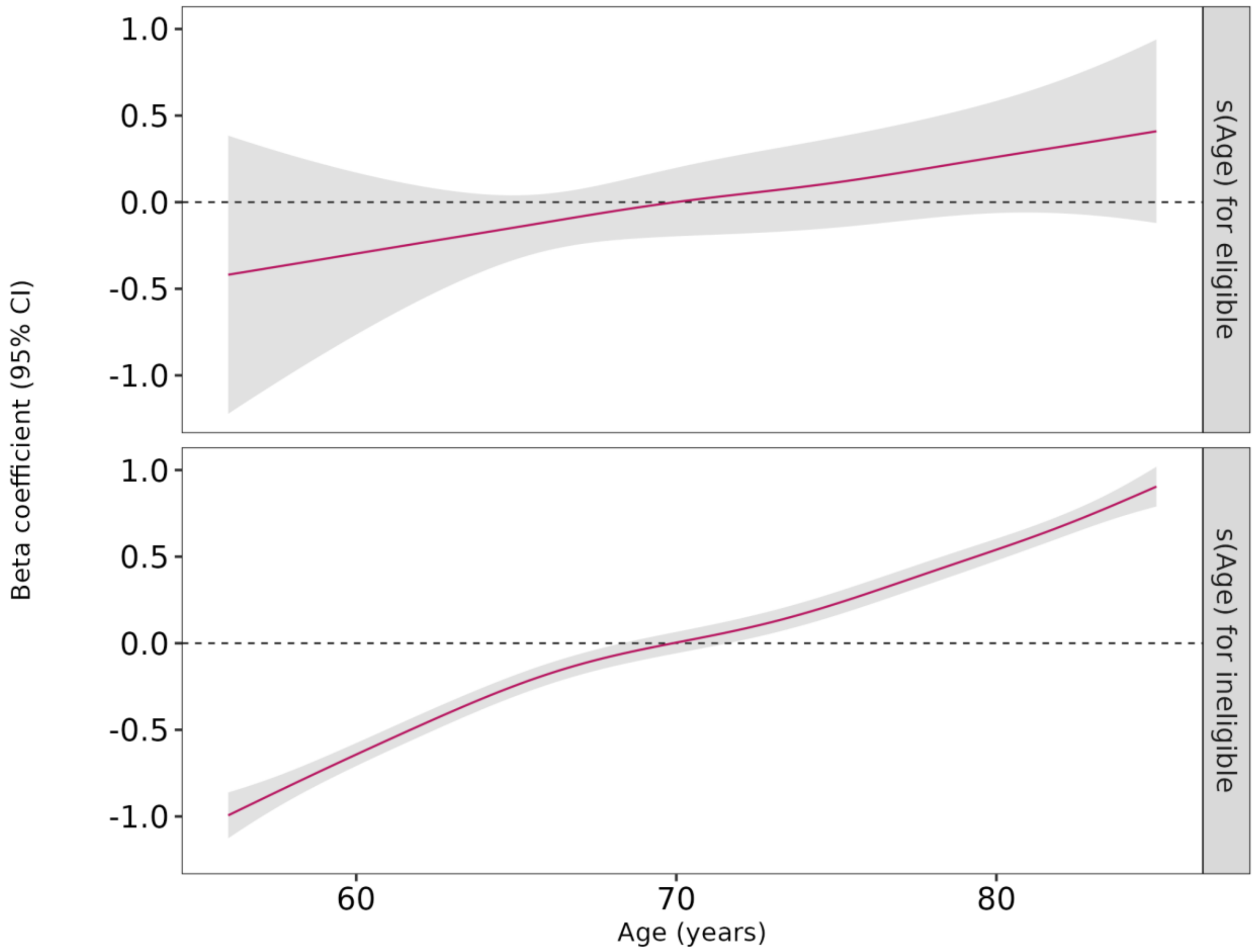

Supplemental figure 3: Modelled relationship between age and the log-incidence of non-invasive pneumococcal pneumonia.

Note that the penalised thin plate regression is modelled such that the beta-coefficient equals zero at the mean age.

Supplemental 6. Observed and predicted number of NIPP cases in the study and national population

Supplemental table 3

The observed and predicted number of non-invasive pneumococcal pneumonia cases in the study population and the extrapolated number in the national population, per epidemiological year

		NIPP				
		Study population		National population		
Birth cohort	Epidemiological year	Observed	With PPV23 [95% CI]	With PPV23 [95% CI]	Without PPV23 [95% CI]	National person-years
Overall	2020-2023	634	635 [586, 690]	1,915 [1,765, 2,079]	2,385 [2,150, 2,648]	5,718,655
1941-1947	2020-2021	66	70 [58, 85]	202 [167, 244]	277 [241, 318]	1,044,586
	2021-2022	126	125 [111, 142]	359 [318, 406]	474 [417, 539]	1,012,166
	2022-2023	151	148 [127, 172]	464 [400, 539]	591 [513, 682]	978,977
1948-1952	2021-2022	105	95 [83, 109]	272 [237, 312]	331 [291, 375]	943,031
	2022-2023	106	113 [101, 127]	355 [316, 400]	411 [355, 475]	927,219
1953-1956	2022-2023	80	83 [69, 99]	259 [217, 310]	299 [261, 343]	812,676

Results were generated with 100,000 parametric Monte Carlo simulations. Note, the epidemiological year includes November up to and including September, i.e. 11 months, as October is excluded. NIPP=non-invasive pneumococcal pneumonia. PPV23=23-valent pneumococcal polysaccharide vaccine. CI=confidence interval.

Supplemental 7. Age-specific impact

Supplemental table 4

Impact estimates per age and time since eligibility for the 23-valent pneumococcal polysaccharide vaccination programme presented in figure 3 of the article

Time since eligibility	Age (years)	Adjusted impact* % [95% CI]
1 year since eligibility	66	10.6 [-16.4, 31.3]
	67	12.8 [-9.2, 30.4]
	68	14.3 [-4.3, 29.5]
	69	15.4 [-1.0, 29.1]
	70	16.3 [1.1, 29.2]
	71	17.4 [2.5, 30.0]
	72	18.8 [3.6, 31.6]
	73	20.6 [4.8, 33.8]
	74	22.8 [6.3, 36.4]
	75	25.1 [7.9, 39.3]
	76	27.6 [9.5, 42.2]
	77	30.0 [10.9, 45.1]
	78	32.3 [12.0, 47.9]
	79	34.4 [12.8, 50.8]
2 years since eligibility	70	10.3 [-11.1, 27.8]
	71	11.5 [-7.5, 27.3]
	72	13.0 [-4.0, 27.3]
	73	15.0 [-0.5, 28.1]
	74	17.3 [3.1, 29.4]
	75	19.8 [6.5, 31.3]
	76	22.5 [9.6, 33.5]
	77	25.0 [12.2, 36.1]
	78	27.4 [14.1, 38.8]
	79	29.7 [15.2, 41.9]
	80	32.0 [15.6, 45.3]
3 years since eligibility	75	14.2 [-9.1, 32.4]
	76	17.0 [-3.9, 33.7]
	77	19.7 [0.7, 35.1]
	78	22.3 [4.6, 36.8]
	79	24.8 [7.4, 38.9]
	80	27.2 [9.3, 41.6]
	81	29.7 [10.3, 44.9]

*=adjusted for time since eligibility, age, and epidemiological year. CI=confidence interval.

Supplemental 8. Number of prevented NIPP cases adjusted for PUAT sensitivity and testing policy

Supplemental table 5

Effect of the pneumococcal urinary antigen test sensitivity and the proportion of patients tested on the number of prevented non-invasive pneumococcal pneumonia in the eligible birth cohorts

		PUAT sensitivity			
		Unadjusted	82.3%	74.0%	66.6%
Tested proportion of patients	Unadjusted	469	570	634	705
	80.0%	587	713	793	881
	70.0%	670	815	906	1,007
	60.0%	782	950	1,057	1,174

The table illustrates the variation in the number of non-invasive pneumococcal pneumonia prevented in the study population when adjusted for different sensitivity values and the proportion of patients suspected of pneumococcal pneumonia who are tested. The numbers were calculated by dividing the number prevented by the product of the two respective values (expressed as decimals). Sensitivity values were based on the systematic review by Sinclair et al.[8] The tested proportion values provided are for illustrative purposes and should not be interpreted as exact measures of the testing policy. Note, that the numbers reflect data obtained for 11 months per epidemiological year. PUAT=pneumococcal urinary antigen test.

Supplemental 9. Leave-one-laboratory-out sensitivity analysis

Excluded laboratory	Cases excluded (%)	Impact [95% CI]
A	428 (11.2)	23.1 [11.2, 33.4]
B	362 (9.5)	15.2 [3.0, 26.0]
C	198 (5.2)	21.1 [9.5, 31.1]
D	295 (7.7)	17.4 [4.7, 28.5]
E	553 (14.5)	16.2 [4.1, 26.9]
F	282 (7.4)	20.7 [9.4, 30.7]
G	178 (4.7)	19.1 [7.8, 29.1]
H	335 (8.8)	19.4 [7.8, 29.6]
I	163 (4.3)	19.9 [8.0, 30.3]
J	324 (8.5)	17.2 [5.1, 27.9]
K	56 (1.5)	20.1 [8.8, 30.1]
L	97 (2.5)	21.8 [10.5, 31.7]
M	302 (7.9)	21.2 [9.5, 31.3]
N	248 (6.5)	24.0 [12.9, 33.8]
Main analysis		19.7 [8.5, 29.7]

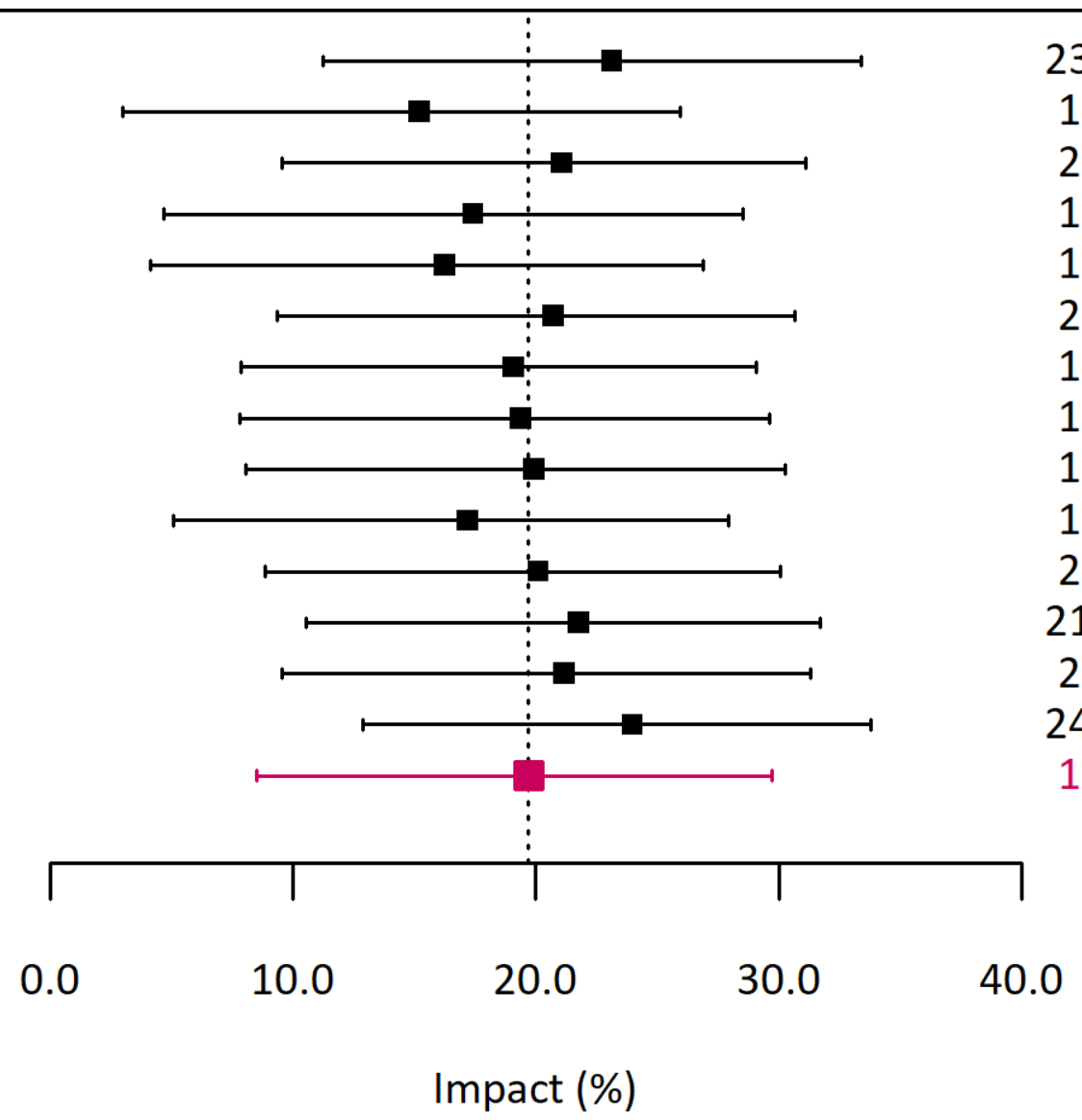

Supplemental figure 4: Impact estimates of the leave-one-laboratory-out sensitivity analysis Impact estimates were calculated as explained before, but one by one, a laboratory was excluded for the analysis. The main analysis includes data of all laboratories. The dashed line represents the point estimate obtained in the main analysis. CI=confidence interval.

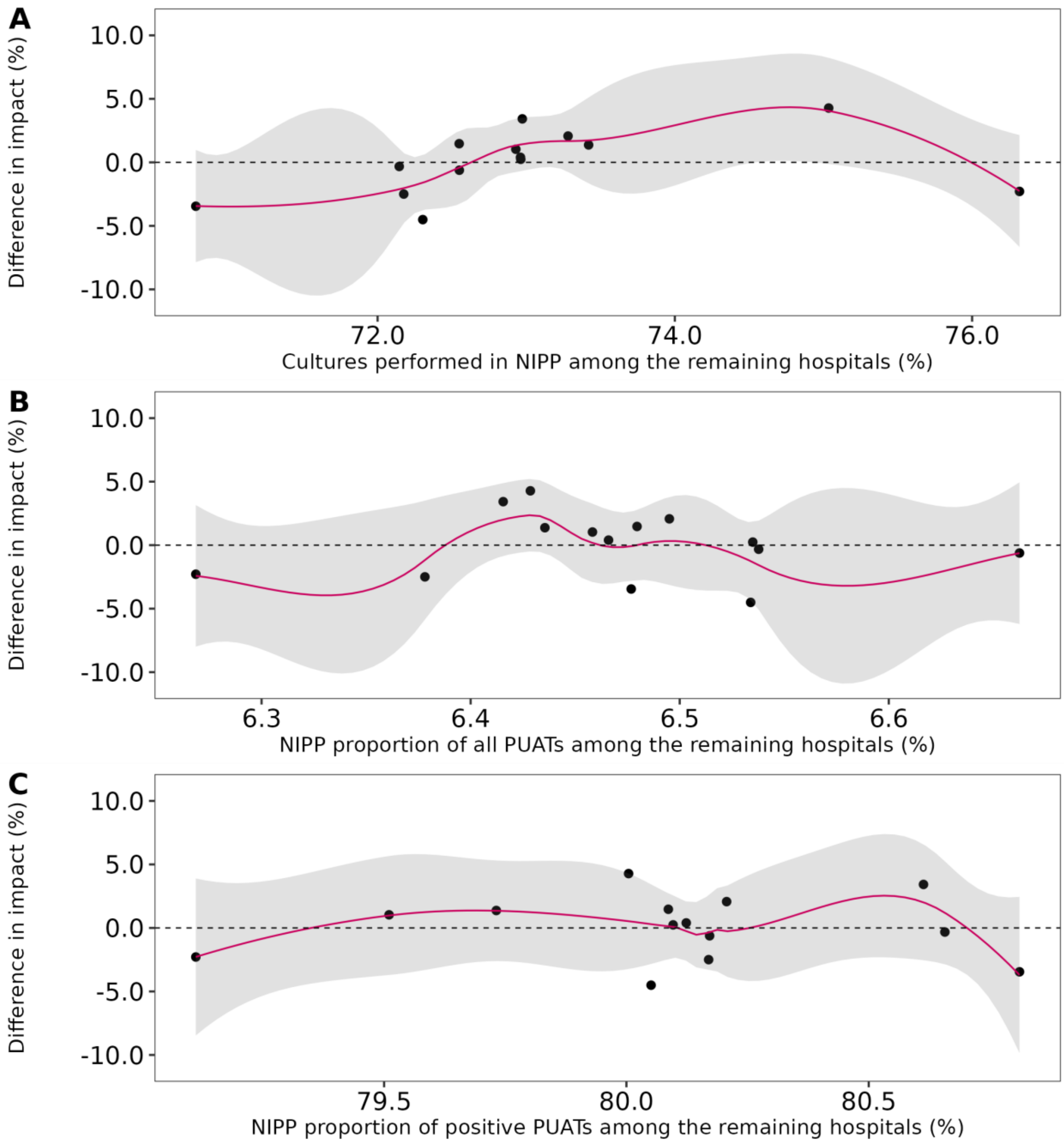

Supplemental figure 5: Difference in impact estimate by proportion of cultures performed in non-invasive pneumococcal pneumonia cases

Each subplot depicts the loess curve for the impact estimate and a characteristic of the remaining laboratories after excluding one. The y-axis represents the difference in impact estimates in the remaining laboratories after excluding one laboratory. Shaded areas represent the 95% confidence intervals. A: the influence of the proportion of NIPP cases in whom culture data was available in a laboratory on the impact estimate (correlation coefficient 0.6, p-value 0.029). Cultures include blood- and/or cerebrospinal fluid cultures. B: the influence of the proportion of NIPP cases among all performed PUATs in a laboratory on the impact estimate (correlation coefficient -0.2, p-value 0.502). C: the influence of the proportion NIPP cases among all positive PUATs in a laboratory on the impact estimate (correlation coefficient -0.1, p-value 0.716). NIPP=non-invasive pneumococcal pneumonia. PUAT=pneumococcal urinary antigen test.

Supplemental references

1. **Statistics Netherlands** Bevolking; geslacht, leeftijd en burgerlijke staat, 1 januari. Available at https://opendata.cbs.nl/#/CBS/nl/dataset/7461bev/table (accessed 10 Jan 2024).
2. **Hahné S, Bollaerts K and Farrington P** (2022) *Vaccination Programmes: Epidemiology, Monitoring, Evaluation*, 1st edn. Abingdon: Routledge.
3. **Oldenburg CE, Moscoe E and Barnighausen T** (2016) Regression Discontinuity for Causal Effect Estimation in Epidemiology. *Curr Epidemiol Rep* **3,** 233–241. https://doi.org/10.1007/s40471-016-0080-x.
4. **Smith LM, Levesque LE, Kaufman JS and Strumpf EC** (2017) Strategies for evaluating the assumptions of the regression discontinuity design: a case study using a human papillomavirus vaccination programme. *Int J Epidemiol* **46**(3)**,** 939–949. https://doi.org/10.1093/ije/dyw195.
5. **van Werkhoven CH and Huijts SM** (2018) Vaccines to Prevent Pneumococcal Community-Acquired Pneumonia. *Clinics in Chest Medicine* **39**(4)**,** 733–752. https://doi.org/10.1016/j.ccm.2018.07.007.
6. **Griens AMGF, Hogewoning SJ, Lukaart JS, Ng CY, Post D and Postma DJ** (2025) Data en feiten 2025, Het jaar 2024 in cijfers. Den Haag: Stichting Farmaceutische Kengetallen. Available at https://www.sfk.nl/sites/sfk-nl/files/2025-09/Data%20en%20feiten%202025%20(1MB).pdf (accessed 06-May-2026).
7. **Zorginstituut Nederland** GIPdatabank.nl: Open Data. Available at https://www.gipdatabank.nl/servicepagina/open-data (accessed 5 Jul 2024).
8. **Sinclair A, Xie X, Teltscher M and Dendukuri N** (2013) Systematic review and meta-analysis of a urine-based pneumococcal antigen test for diagnosis of community-acquired pneumonia caused by Streptococcus pneumoniae. *J Clin Microbiol* **51**(7)**,** 2303–2310. https://doi.org/10.1128/JCM.00137-13.
